# Metabolic Syndrome: Prevalence and Risk Factors among Adolescent Female Intermediate and Secondary Students in Saudi Arabia

**DOI:** 10.3390/ijerph18042142

**Published:** 2021-02-22

**Authors:** Areej Alowfi, Sumayah Binladen, Sumaya Irqsous, Alya Khashoggi, Muhammad Anwar Khan, Ramah Calacattawi

**Affiliations:** 1 Family Medicine & Primary Health Care Department, Ministry of the National Guard—Health Affairs, Jeddah 21423, Saudi Arabia; 2 College of Medicine, King Saud Bin Abdulaziz University for Health Sciences, Jeddah 21423, Saudi Arabia; Binladen001@ksau-hs.edu.sa (S.B.); Airqsous024@ksau-hs.edu.sa (S.I.); Khashoggi002@ksau-hs.edu.sa (A.K.); khana@ksau-hs.edu.sa (M.A.K.); calacattawiRa@ngha.med.sa (R.C.); 3 King Abdullah International Medical Research Center, Jeddah 21423, Saudi Arabia

**Keywords:** metabolic syndrome, obesity, overweight, Saudi Arabia, adolescents, female, school-aged, hyperlipidemia, hypertension, risk, prevalence

## Abstract

Background: Metabolic syndrome (MS) has become one of the major challenges to public health worldwide due to its significant association with increased risk of developing type 2 diabetes and cardiovascular disease (CVD) among children and adolescents. Therefore, this study aims to determine the prevalence and risk factors of MS in Saudi adolescents. Methods: This cross-sectional study was conducted in two female National Guard schools (Um Kalthoom Intermediate School and Zainab Bint Jahsh Secondary School) in Jeddah, Saudi Arabia, between January 2018 and March 2018. Of the 808 female students, 172 (age range of 12–19 years) participated voluntarily, with consent from their guardian(s), fasted for at least 8 h prior to the study, and represent the final study sample. Male students were not included due to cultural constraints in conducting the study. Demographic data, physical measurement (blood pressure, weight, height, body mass index (BMI) and waist circumference (WC)), and biochemical measurement (fasting plasma glucose and triglycerides, high-density lipoprotein (HDL), and low-density lipoprotein) were obtained. The International Diabetes Federation (IDF) criteria was utilized in the diagnosis of MS. Results: Of the 172 female adolescents, 24 (13.75%) are overweight, 20 (11.63%) are obese, and 3 (1.74%) are underweight. High-fasting plasma glucose (*n* = 85, 49.41%) and high waist circumference (*n* = 74, 43.02%) were the most common risk factors of MS among female adolescents. The overall prevalence of MS was 7% (*n* = 12/172). MS is more common among those who are obese or among those with a BMI that falls at the ≥95th percentile (*n* = 6/20, 30.0%) (*p* < 0.05) as compared to those who are overweight with a BMI that falls within the 85th to <95th percentiles (*n* = 2/24, 8.33%). Interestingly, 3.20% of the sample (*n* = 4/125) with normal BMI were diagnosed with MS. Conclusions: Our study indicates that MS is common among obese and overweight female adolescents but is also present among those who are not obese or with normal BMI. Moreover, the prevalence of overweight, obese, and those with MS in this reference population are lower compared to the cities of Makkah and Riyadh; however, it varies widely around the world due to different criteria and cut-off values in the diagnosis of MS.

## 1. Introduction

Metabolic syndrome (MS) has become one of the major challenges to public health worldwide due to its significant association with an increased risk of developing type 2 diabetes and cardiovascular disease among children, adolescents, and adults [1]. A diagnosis of MS is defined as the presence of abdominal obesity with two or more risk factors, such as high fasting triglycerides (TG), low high-density lipoprotein (HDL) cholesterol, elevated blood pressure (BP), and high fasting plasma glucose (FPG) [2]. For example, an individual with abdominal obesity, along with elevated blood pressure and high FPG has an increased risk of cardiovascular disease (CVD) because of the combination of the risk factors than any factor presenting alone [3]. A prospective cohort study including 6255 subjects indicated that those having one or two MS risk factors were at increased risk for mortality from coronary heart disease (CHD) and (CVD). The study also indicated that MS strongly predicts CHD, CVD, and total mortality as compared to its individual components [4].

According to the third United States National Health and Nutrition Examination Survey (NHANES III), MS is becoming increasingly common among children and adolescents particularly in obese and overweight populations [5]. The International Obesity Task Force (IOTF) estimated that approximately 10% of school-aged children (i.e., aged 5–17 years) worldwide—representing 155 million children—are obese and overweight [6].

The prevalence of MS in children varies widely worldwide and because of the different criteria used in diagnosing MS, including the National Cholesterol Education Program’s Adult Treatment Panel III (ATP III), International Diabetes Federation (IDF), and World Health Organization (WHO) [2,7,8,9]. In the systematic review of 463 studies describing the epidemiology of MS in children, the median MS prevalence was 3.3% (0–19.2%), in which 11.9% (2.8–29.3%) was found in overweight populations and 29.2% (10.0–66.0%) was found in obese populations [7].

In Saudi Arabia, one of the most rapidly emerging metabolic disorders in children is obesity. In 2010, the WHO reported that the prevalence of obesity and overweight in Saudi children aged 5–19 years were 14.3% and 31.6%, respectively, and increased in 2016 to 17.4% and 35.6%, respectively [10]. In a study conducted in the city of Tabuk among children aged 6–13 years, the prevalence of obesity and overweight among males was 17.4% and 7.3%, respectively, while it was 20.9% and 12.4% among females, respectively [11]. In another study on 7930 children aged 6–16 years in the city of Riyadh, the prevalence of obesity and overweight for males was 18.4% and 12%, respectively, while it was 18% and 14.2% for females, respectively [12].

Although the prevalence of MS among adults in Saudi Arabia (i.e., those ≥18 years old) has been reported to be approximately 31.6% (IDF criteria) and 39.9% (ATP III criteria) [13], little is known regard to the prevalence of MS in children and adolescents particularly in a school setting and in the city of Jeddah, Saudi Arabia. Moreover, sample population in several studies were clustered according to age duration or/and not according to the developmental age group in which the result of prevalence of MS in every age group particularly in children and adolescents cannot be identified [14,15]. In the meta-analysis published recently to determine the prevalence of MS in the country, of the 19 selected studies conducted between 2005 and 2019 including 37,745 subjects for analysis, the 16 studies were conducted among the sample population aged 20 years and above, 2 studies conducted among those aged 18 and above (i.e., the adolescent age was clustered with adult age group), and 1 study conducted among children and adolescents but age group was not categorized accordingly [16].

Therefore, this study aims to estimate the prevalence of MS among adolescent (i.e., aged 12–19 years) female intermediate and secondary students in the city of Jeddah, Saudi Arabia and to determine the most common MS risk factors in this reference population. The results of this study will serve as a basis in order to establish a plan for developing effective interventional programs for the prevention of chronic diseases in adolescents, such as diabetes mellitus and CVD.

## 2. Materials and Methods

This cross-sectional study was conducted in two female National Guard schools (Um Kalthoom Intermediate School and Zainab Bint Jahsh Secondary School) in Jeddah, Saudi Arabia, between January 2018 and March 2018, to measure the prevalence of MS and MS risk factors among female adolescents aged 12–19 years.

Of the 808 female students, 273 were voluntary participants and deemed eligible for the study; however, only 172 fasted for at least 8 h and represent the final study sample. Male students were not included in the study due to cultural constraints in obtaining permission to conduct the study since all researchers and medical practitioners who collected data and obtained blood samples were female. In addition, students who were not in the age range of 12–19 years were excluded.

In the data collection for the study, a structured questionnaire was created on the basis of the WHO STEPwise approach to chronic disease risk factor surveillance (STEPS). The STEPS instrument encompasses three different levels or “steps” of risk factor assessment: use of a questionnaire, physical measurements, and biochemical measurements [17]. The questionnaire consists of certain items to determine students’ demographic data (i.e., age and school year level), parental history of chronic diseases (e.g., diabetes mellitus, hypertension, hyperlipidemia, and obesity), diet, and physical activities. In terms of physical measurements, blood pressure, weight, height, body mass index (BMI), and waist circumference (WC) of students were obtained. All measurements were performed out on school premises between 8:00 a.m. and 10:00 a.m. in a quiet room with adequate lighting. Upon the arrival of the students in the designated rooms, they were asked to rest in a seated position for five minutes before their blood pressure was assessed using a portable automated blood pressure device (Omron BP M6 comfort, Omron Healthcare Ltd., Kyoto, Japan). This instrument is equipped with a single and repeated measure function, which measures blood pressure three times and displays a calculated average value. Appropriate cuff sizes were used based on the size of the student’s arm. The cuff was wrapped around the upper left arm and maintained in place with Velcro. Blood pressure is determined automatically. Further, height was measured to the nearest 0.1 cm without shoes, using a Stadiometer with the shoulders in a relaxed position. Weight was measured standing (without shoes) using a mechanical beam scale. BMI was calculated by dividing weight (kg) to height in square meters (m^2^) and categorized into underweight (<5th percentile), normal (5th to <85th percentile), overweight (85th to <95th percentile), and obese (≥95th percentile) based from the growth chart for Saudi children and adolescents aged 5–19 years [18,19]. WC was measured to the nearest 0.1 cm at the smallest point between the iliac crest and the lower rib margin at the end of normal expiration, using a non-elastic tape with participants standing with both feet together. Lastly, biochemical measurements—including fasting plasma glucose, lipid profiles (triglycerides, high-density lipoprotein (HDL), and low-density lipoprotein-cholesterol)—were also obtained using the CardioChek PA Analyzer (Polymer Technology Systems, Inc. CardioChek, Zionsville Rd, IN, USA) that has been proven to be as reliable as hospital lab sampling [20].

Written informed consent and verbal consent were obtained from school authorities, guardians, and students prior to both data and blood sample collection. The ethical approval of the study was obtained from King Abdullah International Medical Research Center (KAIMRC) (IRB Ref. no. SP17/279/J).

In order to identify female students with MS, we adopted the International Diabetes Federation (IDF) criteria. The student must have abdominal obesity or WC of ≥80 cm, and two or more risk factors (i.e., triglycerides level of ≥150 milligrams per deciliter of blood (mg/dL), HDL cholesterol of <50 mg/dL, systolic blood pressure (SBP) of ≥130 mm of mercury (mmHg), diastolic blood pressure (DBP) of ≥85 mmHg, or FPG of ≥100 mg/dL). Because no reference values exist for WC for Saudi children and adolescents, we adopted the recommendation proposed by the IDF for WC threshold of ≥80 cm [2,21].

All data collected were analyzed using the Statistical Package for Social Sciences (SPSS), version 20.0 (IBM Corp., Armonk, NY, USA). The data were presented in mean and standard deviation for continuous variables and frequencies and percentages for categorical variables. Comparisons among groups were made using appropriate inferential tests such as Student *t*-test, Chi-square test, and ANOVA. When group differences were found, a post-hoc analysis was performed. The level of significance was set at a *p*-value of <0.05.

## 3. Results

Of the 172 female adolescents (aged 12–19 years), 125 have normal BMI, 24 are overweight, 20 are obese and 3 are underweight, respectively. Obese (≥95th percentile) adolescents had a significantly higher WC, weight, and BMI. They are also relatively shorter in height, have higher blood pressures, higher triglyceride levels, lower HDL levels and higher LDL levels as compared to adolescents with normal BMI. Surprisingly, adolescents with normal BMI had a higher fasting plasma glucose level. The difference in baseline physical characteristics and MS variables of the subjects is presented in Table 1.

Overall, high glucose levels and high WC were the most common among female adolescents, whereas low HDL and high triglycerides were the least common. Females aged 12–15 years had a higher percentage of high glucose level and high triglycerides, whereas aged 16–19 years had a higher percentage of high WC, elevated blood pressure, and low HDL. Obese adolescents significantly had the highest proportion of high WC, elevated blood pressure, and low HDL level. High glucose also is commonly observed among obese adolescents but not statistically significant. Further, adolescents with normal BMI had the highest rate of high triglycerides level, but this was not statistically significant. For three underweight adolescents, one had high WC, and two had high blood glucose levels. The distribution of prevalence of individual MS risk factors is presented in Table 2.

**Figure 1:**
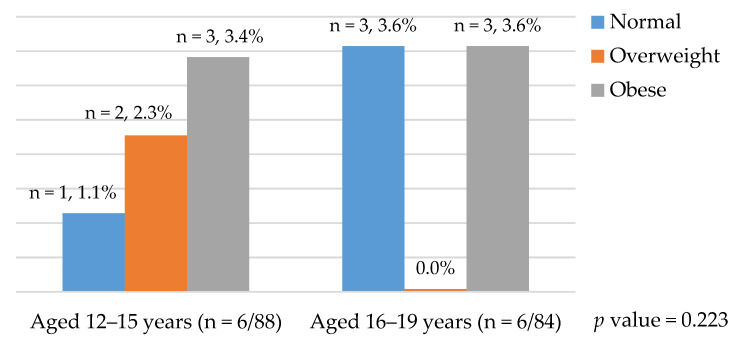
Prevalence of metabolic syndrome (MS) by age and body mass index (BMI).

The overall prevalence of MS among female Saudi adolescents (aged 12–19 years) was 7% and one of them was diagnosed with Type II diabetes mellitus. MS is more common among obese or among those with a BMI that falls in the ≥95th percentile (30.0%) (*p* < 0.05) as compared to those who are overweight or with a BMI that falls within the 85th to <95th percentiles (8.33%). Adolescents with normal BMI also have an MS prevalence of 3.20%. One in the age group of 12–15 years and three in the age group of 16–19 years. The prevalence of MS among those in the age group of 16–19 years of age and 12–15 years of age was no significant difference (*p* > 0.05) (Table 3). Figure 1 indicates that there is an incremental increase in the prevalence of MS when age and BMI increase. The prevalence of MS by age and BMI are presented in Figure 1. Adolescents with parental history of diabetes mellitus, hypertension, obesity, hyperlipidemia, or high cholesterol level have a higher prevalence of MS, but not statistically significant. Those who eat fast-food often and do no weekly exercise have a higher prevalence of MS, but this is not statistically significant (Table 3).

Of the 172 adolescents, 35 (20.35%) are healthy or without the presence of any of the risk factors, while 137 (79.65%) have at least ≥1 risk factor. Most of the adolescents had ≤2 MS risk factors (29.08%), whereas 7.46% had >2 risk factors. Further, in post hoc analysis, adolescents with high WC had a significantly higher frequency of risk factors (i.e., ≥2 risk factors, 64.87%); however, adolescents with normal WC had a significantly higher frequency of one risk factor (57.14%). All obese adolescents had at least one MS risk factor. In addition, obese adolescents also had more risk factors (≥2 risk factors, 70.00%) followed by overweight ones (50.00%); however, adolescents with normal BMI had a higher frequency of one risk factor (50.40%). It is noteworthy that all three underweight adolescents have 1 MS risk factor (i.e., n = 1, high waist circumference and n = 2, high fasting plasma glucose level). In post hoc analysis obese adolescents had a significantly higher frequency of four risk factors as compared to adolescents with normal BMI (Table 4).

## 4. Discussion

Rapid urbanization, unhealthy diets, and increasingly sedentary lifestyles of populations globally have made obesity an emerging pandemic. This serious public health problem increases the incidence of MS accompanied by a variety of conditions, such as hypertension, hypertriglyceridemia, hypercholesterolemia, and high glucose level.

The massive transformation in Saudi Arabia’s economic and health care sectors at the beginning of the twenty-first century resulted in an increase in lifestyle diseases, such as obesity, diabetes, and cardiovascular diseases. In fact, the country is one of the top countries worldwide with a high prevalence of diabetes and rate of obesity, with the other components of MS also significantly increasing [22].

The intent of the study is to determine the prevalence of MS among children and adolescents in Saudi Arabia; in Jeddah this was revealed to be 7%, which is lower compared to Makkah (17.1%) [23] and Riyadh (2% to 18%) [24]; however, when compared with the result of the study done in Riyadh [24] using the same criteria (i.e., IDF criteria), our result is higher because the participants of this study were of pubertal age and were relatively older. Moreover, in their study, they included students with the age of 11 years and below. When compared with other countries, United Arab Emirates has 3.7% [25], Chile has 9.5% [26], and South Korea has 2.3% [27]. In addition, in a systemic review, the prevalence of MS was reported to be 3.3% [7]. This could be due to numerous factors, one being the wide variety of diagnostic criteria used to define MS [2,7,8,9]. Moreover, another factor influencing the prevalence of MS to be higher than that found in other studies may be due to the fact that this study was conducted on a specific demographic (female children and adolescents only) where all other studies included both males and females. It is suggestive that MS could be more prevalent in the female population. This was reported in a study conducted in India where MS was found to be more prevalent in girls than boys [28]. Another study contradicted this finding reporting that the prevalence of MS was higher among boys than in girls [25]. The conflicting findings based on gender are suggestive of the fact that gender may not be a significant predictor when it comes to MS. Other factors could be differences in patient selection, ethnicity, and age range.

In terms of BMI, the prevalence of overweight and obese among female students in this study is 13.95% and 11.63%, respectively, which is lower than that in studies conducted in Makkah (15.2% overweight and 15.3% obese) [23] and in Riyadh (21.9% overweight and 20.6% obese) [29]. It can be inferred that the location of the studies previously mentioned is all metropolitan cities of the country. It is then important to consider the regional differences and the subjects’ place of residence, since there are cultural differences, which—in one way or the other—may affect the dietary habits and lifestyle of the people. El Mouzan et al. [30] compared the prevalence of obesity and overweight in central (Riyadh and Qassim), southwest (Jizan and Asir), and northern (Hail, Jouf, and Northern Borders) regions. A lower prevalence of obesity and overweight in the southwest regions compared to central and northern regions was noted; there were no significant differences between central and northern regions in obesity and overweight rates. Similarly, Al-Hazzaa et al. [31] explored the differences among three regions (Riyadh, Makkah, and Eastern Regions) and revealed that all these cities are urban and highly populated. Even though no significant differences in the prevalence of obesity and overweight exists among the three cities, abdominal obesity (assessed by WC and waist-to-hip ratio) was different among adolescents (14–19 years) from these cities; post hoc analyses were not conducted, but abdominal obesity appeared to be highest among females in Jeddah (Makkah region).

Numerous studies have also reported the difference in the prevalence of MS that affected both urban and rural populations with different patterns of MS combinations. Normally, it is expected that children who live in urban areas tend to have a higher risk of obesity and overweight, which can be attributed to unhealthy dietary behaviors, like high intake of sugar-sweetened beverages [32,33,34,35,36,37,38,39].

However, surprisingly, the meta-analysis of 74,168 pooled participants in the age group 2–19 years reported that rural children have 26% greater odds of obesity compared to urban children in the United States [40]. Another study in Korea similarly noted a much higher MS prevalence in rural (39.8%) than that in urban (22.5%) subjects (*p* < 0.001). Those residing at the rural areas showed significantly higher blood pressure (*p* < 0.001), serum triglyceride levels (*p* < 0.001), and LDL (low density lipoprotein)-cholesterol level (*p* < 0.001), as well as the odds ratio (OR) for MS (OR = 1.65, 95% CI: 1.59–1.71), as compared to urban residents [41]. Similarly, this fact was confirmed by other similar studies [33,42,43,44]. Further, the prevalence of metabolic syndrome was also reported as being higher in rural than in urban residents (39.9% vs. 32.8%), among both men (39.7% vs. 33.3%) and women (40.2% vs. 32.3%, respectively) [45].

In contrast, a few studies revealed the prevalence of obesity and overweight to be typically higher in urban areas, while the incidence of underweight is typically higher in rural areas [46,47,48]. Further, a national survey in 33 Indonesian provinces calculated the odds ratio (OR) adjusted with all variables (such as age, gender, residency, education level, physical activity, and food intake) and revealed that there exists an urban-rural difference in the factors related to obesity among children and adolescents. It indicated that the risk of obesity and overweight in urban areas was related to daily caffeinated soft drinks and consumption of energy drinks (OR = 1.12, 95% CI: 1.01–1.23). The daily consumption of grilled foods (OR = 1.32, 95% CI: 1.22–1.42) and salty foods (OR = 1.09, 95% CI: 1.04–1.15) was significantly associated with obesity in rural areas but not in urban areas [49].

Further, sedentary activity was correlated with obesity and overweight among both urban and rural residents, thereby necessitating the need to focus initiatives for education, environmental, and policy interventions owing to better access to a wide variety of processed and traditional high-sugar, high-fat snack foods, and beverages [40]. A study conducted among residents in Eastern China has the same findings—residing in urban areas was associated with higher dietary fat intake and slightly lower total energy intake and with significantly lower occupational physical activity [50].

Numerous studies also suggested that the prevalence of MS has also been seen among children and adolescents in Saudi Arabia. Despite the difficulty in estimating the prevalence of MS in children due to the different criteria used in its multiple definitions worldwide, there is a rising prevalence of obese Saudi children and adolescents having multiple risk factors associated with metabolic syndrome [51]. A similar trend in the other parts of the globe was noted. The median prevalence of MS in entire populations was 3.3% (range 0–19.2%), 11.9% (range 2.8–29.3%) among overweight children, and 29.2% (range 10–66%) among obese populations. For non-obese, non-overweight populations, the range was 0–1%. This was part of a systematic review of 85 studies in children [7]. Current estimates also indicate that approximately 4% of US adolescents have a metabolic syndrome, based on the results of a previous study [5]. A recent study indicated a prevalence of 6.8% among US college students aged 18 to 24 years [52].

Corroborating with other studies, our results revealed that high glucose levels followed by high waist circumference were common among Saudi female adolescents under study, as also reported by similar studies conducted in the country. Abolfotouh et al. [29] reported that central adiposity among college students contributes to the high incidence of individual MS components. Similarly, Al-Hazzaa et al. [31] revealed that the prevalence of abdominal obesity in males and females was 35.9% and 30.3%, respectively. A higher prevalence of obesity was observed among adolescents in private schools.

Further, factor analysis was utilized by certain studies to examine MS among adolescents and adults, but they failed to find a single factor contributing to MS, thereby indicating that the number of factors and their loading patterns differs depending on the baseline characteristics of the studied populations and the variables included in the analysis [53,54]. Factor analysis revealed that BMI/insulin/lipids, BMI/insulin/glucose, and blood pressure—with a unifying role for markers of insulin resistance and adiposity—underlie the MS among the Canadian youth [55]. The study by Goodman et al. [56] among US adolescents found that adiposity, cholesterol, and carbohydrate/metabolic factor are the three factors correlated with MS. Similarly, he considered obesity as the predominant correlate of coronary artery disease risk factors, thereby emphasizing that BMI and obesity are associated with every risk factor measured.

On the other hand, Cruz et al. [57] defined the pediatric metabolic syndrome as the presence of at least three of the following: abdominal obesity (WC ≥ 90th percentile), low HDL-C level (≤40 mg/dL), hypertriglyceridemia (>90th percentile), hypertension (>90th percentile), and/or impaired glucose tolerance in another US study. Kashayar et al. [58] measured dyslipidemia, high fasting plasma glucose concentrations, and high blood pressure values in order to determine the presence of MS.

It is also important to note that the other studies conducted in other parts of the world found a significant association between age and gender with the MS. A few studies also reported that MS is more prevalent among girls, while other studies have presented opposing viewpoints. In fact, in Latin America, 32.2% of girls compared to 40% of boys aged 5–18 years had MS [59]. Similarly, Agirbasli et al. [60] reported the condition to be more common among Turkish boys.

Similar to the results of the study by Kashayar et al. [58], Ferreira et al. [61] classified 10.7% of boys and 25% of girls with MS based on the NCEP ATP III diagnostic criteria. The main underlying factor contributing to the higher numbers of girls diagnosed with MS in studies can be attributed to hormonal changes and subsequent central body fat accumulation, particularly during puberty [62].

It must also be noted that obese adolescents in this study are significantly higher in four MS risk factors, except for FPG. Their FPG levels were high but not statistically significant. On the contrary, those who were underweight were found to have one MS risk factor, usually high WC and high glucose level. A study conducted in Palestine that investigated the prevalence of individual MS components according to the IDF and National Cholesterol Education Program Adult Treatment Panel (NCEP) criteria reported 32% and 25.3% for increased WC, 15.8% and 37.0% for increased blood pressure, 9.7% and 24.8% for increased triglyceride, 57.2% and 55.9% for low HDL, and 39.7% and 15.8% for increased fast blood sugar, respectively. It can be emphasized that clustering of metabolic abnormalities was found to significantly increase with increasing BMI and WC and with decreased HDL and elevated triglyceride [63].

Khashayar et al. [58] investigated the prevalence of different combinations of the risk factors leading to MS among a nationally representative sample of adolescents in the Middle East and North Africa (MENA) and found that a low HDL-C was the most common component (43.2% among the overweight/obese versus 34.9% of the normal-weight participants), whereas high blood pressure was the least common component. The study has shown alarming evidence-based data on the considerable prevalence of obesity, MS, and CVD risk factors in the adolescent age group in the MENA. These results further serve as confirmatory evidence for the clear calls for action for prioritizing the importance of primordial/primary prevention of noncommunicable disease, particularly in communities facing a double burden of nutritional disorders.

Further, the younger population (aged 12–15 years) in this study have high glucose levels and triglycerides; a high WC, high blood pressure, and low HDL were noted among older children (aged 16–19 years). Indeed, the rising trend of childhood obesity prevalence over the past three decades in Saudi Arabia has led to adult morbidity and obesity, thereby causing several adverse health, social, and economic outcomes [45]. The predisposition to diseases such as diabetes mellitus and CVD is brought about by the following MS risk factors: hypertension, hypertriglyceridemia, hypercholesterolemia, and high glucose level. It must be noted that these factors are also regarded as important elements of increased cardiometabolic risk in addition to abdominal obesity, smoking, insulin resistance, high blood pressure, high low-density lipoprotein cholesterol (LDL-C), low high-density lipoprotein cholesterol (HDL-C), high triglycerides (TG), high fasting plasma glucose, and disturbed inflammatory profile. In order to decrease the disease burden of MS among children and adolescents in subsequent years, weight loss and lifestyle modifications must then be the main areas for MS treatment and prevention, particularly in schools.

Our study has several limitations: a small sample size to represent the adolescent population in the city of Jeddah, the lack of determination of the socioeconomic status of the family, certain contributing factors or extraneous variables of MS were not assessed to determine the cause of MS among adolescents, and there was no sample to represent students from private schools for comparison. These limitations of the present study will be addressed in our future studies in order to increase the sample size to encompass both public and private schools and compare the prevalence of MS between males and females.

## 5. Conclusions

The prevalence rate of MS among Saudi Female adolescents as revealed in this present study is 7%. Our data findings have revealed that MS is common among those who are obese and overweight; however, it is also found in those who are non-obese or with normal BMI. High glucose levels and high waist circumference were the most common MS risks factors identified among female adolescents. This study also reveals that the prevalence of overweight, obese, and MS in this reference population is lower compared to that in the cities of Makkah and Riyadh; however, these variables vary widely around the world due to different criteria and cut-off values in the diagnosis of MS.

## Figures and Tables

**Table 1:** Physical characteristics and MS variables differences when stratified according to age and BMI.

Variables	Age			BMI				
	12–15 *n* = 88	16–19 *n* = 84	*p* Value	Underweight *n* = 3	Normal *n* = 125	Overweight *n* = 24	Obese *n* = 20	*p* Value
Waist circumference (cm)	78.15 (12.71)	78.92 (12.71)	0.680	68.33 (14.04)	74.23 (8.43)	88.79 (8.29)	94.55 (13.85)	0.000 *
Weight (kg)	55.40 (11.02)	55.99 (16.87)	0.786	36.00 (8.00)	50.47 (7.81)	65.10 (5.68)	80.00 (19.55)	0.000 *
Height (cm)	154.35 (5.69)	152.21 (14.53)	0.203	152.67 (14.74)	154.16 (6.88)	155.04 (5.25)	145.93 (25.49)	0.269
BMI (kg/m^2)^	23.13 (4.30)	23.96 (8.02)	0.396	15.31 (.98)	21.12 (2.49)	27.07 (1.80)	35.65 (10.14)	0.000 *
Systolic Blood Pressure (mmHg)	107.35 (11.43)	107.49 (14.39)	0.941	107.0 (9.54)	106.35 (13.53)	107.08 (11.15)	114.50 (9.34)	0.075
Diastolic Blood Pressure (mmHg)	75.22 (10.55)	75.38 (11.18)	0.921	81.00 (2.65)	74.05 (11.55)	77.20 (9.38)	79.90 (6.01)	0.076
Fasting Plasma Glucose (mg/dL)	100.69 (12.35)	105.77 (39.83)	0.256	96.67 (18.93)	104.68 (33.29)	99.00 (13.69)	99.75 (11.53)	0.746
Triglycerides (mg/dL)	89.36 (40.32)	88.37 (41.52)	0.874	69.67 (4.16)	88.10 (43.58)	86.50 (29.09)	99.45 (36.99)	0.548
High-Density Lipoprotein (mg/dL)	55.13 (12.06)	52.44 (12.96)	0.161	48.00 (3.00)	54.46 (11.75)	53.45 (14.93)	51.05 (14.97)	0.581
Low-Density Lipoprotein (mg/dL)	59.90 (23.32)	61.55 (25.89)	0.661	49.67 (23.80)	59.58 (24.43)	66.17 (18.98)	62.85 (31.00)	0.533

Underweight, <5th Percentile; Normal, 5th to <85th percentile; Overweight, 85th to <95th percentile; Obese, ≥95th percentile; All data are presented in Mean (SD). * Post hoc test mean differences were significant between normal, overweight, and obese. The *p*-values of the post hoc comparisons for mean waist circumference and weight were Bonferroni-adjusted. BMI body mass index.

**Table 2:** Prevalence of individual Metabolic Syndrome risk factors among female adolescents aged 12–19 years.

Variables	Elevated WC *n* (%)	*p*	Elevated BP *n* (%)	*p*	High FPG *n* (%)	*p*	High TG *n* (%)	*p*	Low HDL *n* (%)	*p*
Total (*n* = 172)	74 (43.02%)		25 (14.53%)		85 (49.41%)		9 (5.23%)		17 (9.88%)	
Age										
12–15 (*n* = 88)	37 (42.00%)	0.791	12 (13.64%)	0.732	45 (51.14%)	0.645	5 (5.68%)	0.787	8 (9.09%)	0.721
16–19 (*n* = 84)	37 (44.04%)		13 (15.48%)		40 (47.62%)		4 (4.76%)		9 (10.71%)	
BMI										
Normal (*n* = 125)	34 (27.20%) *	0.000	15 (12.00%) *	0.046	62 (49.60%)	0.757	7 (5.60%)	0.968	9 (7.20%) *	0.015
Underweight (*n* = 3)	1 (33.33%)		0 (0.00%)		2 (66.67%)		0 (0.00%)		0 (0.00%)	
Overweight (*n* = 24)	21 (87.50%) *		3 (12.50%)		10 (41.67%)		1 (4.16%)		2 (8.33%)	
Obese (*n* = 20)	18 (90.00%) *		7 (35.00%) *		11 (55.00%)		1 (5.00%)		6 (20.00%) *	

All data are presented by frequency and percentage. * Post hoc test for differences in proportions between groups were significant. The *p*-values of the post hoc comparisons for BMI were Bonferroni-adjusted. WC waist circumference, BP blood pressure, FPG fasting plasma glucose, TG triglycerides, HDL high density lipoprotein, BMI body mass index.

**Table 3:** Prevalence of Metabolic Syndrome among female Saudi Adolescents aged 12–19 years by demographic and dietary habits.

Variables	No. of Samples	Number of Adolescents with Metabolic Syndrome *n* (%)	*p*-Value
Total	172	12 (7.0%)	
Age (years) 12–15	88 (51.16%))	6 (6.89%)	0.933
16–19	84 (48.84%)	6 (7.14%)	
BMI underweight (<5th Percentile)	3 (1.74%)	0 (0.0%)	0.000
Normal (5th to <85th percentile)	125 (72.68%)	4 (3.20%) *	
Overweight (85th to <95th percentile)	24 (13.95%)	2 (8.33%)	
Obese (≥95th percentile)	20 (11.63%)	6 (30.0%) *	
Parental history of diabetes mellitus			
Yes	40 (23.26%)	5 (12.5%)	0.118
No	132 (76.74%)	7 (5.30%)	
Parental history of hypertension			
Yes	50 (29.07%)	5 (10%)	0.319
No	122 (70.93%)	7 (5.74%)	
Parental history of high cholesterol level			
Yes	34 (19.77%)	4 (11.76%)	0.221
No	138 (80.23%)	8 (5.80%)	
Parental obesity			
Yes	26	2 (7.69%)	0.876
No	146	10 (6.85%)	
Fast food consumption (daily)			
No	16	1 (6.25%)	0.609
Sometimes	115	10 (8.70%)	
Always	40	1 (2.5%)	
Weekly exercise			
Yes	71	4 (5.63%)	0.562
No	101	8 (7.92%)	

* Post hoc test for differences in proportions between BMI groups were significant. The *p*-values of the post hoc comparisons for BMI were Bonferroni-adjusted. BMI body mass index.

**Table 4:** Prevalence of the number of MS risk factors by age, waist circumference, and BMI categories.

Variables	0 Risk Factors	1 Risk Factor	2 Risk Factors	3 Risk Factors	4 Risk Factors	5 Risk Factors	*p*-Value
Total (*n* = 172)	35 (20.35%)	82 (4.67%)	42 (24.41%)	9 (5.23%)	4 (2.33%)	0 (0.00%)	
Age 12–15 (*n* = 88)	20 (22.73%)	44 (50.00%)	23 (26.14%)	6 (6.82%)	3 (3.41%)	0 (0.00%)	0.487
16–19 (*n* = 84)	15 (17.86%)	38 (45.24%)	19 (22.62%)	3 (3.57%)	1 (1.19%)	0 (0.00%)	
Waist circumference							
Normal (<80 cm) (*n* = 98)	35 (35.71%)	56 (57.14%) *	7 (0.00%)	0 (0.00%)	0 (0.00%)	0 (0.00%)	0.000
High (≥80 cm) (*n* = 74)	0 (0.00%)	26 (35.14%)	35 (47.30%) *	9 (12.16%) *	4 (5.41%) *	0 (0.00%)	
BMI							
Normal (*n* = 125)	33 (26.40%)	63 (50.40%)	25 (20.00%)	3 (2.40%)	1 (0.80%)	0 (0.00%)	0.000
Underweight (*n* = 3)	0 (0.00%)	3 (100%)	0 (0.00%)	0 (0.00%)	0 (0.00%)	0 (0.00%)	
Overweight (*n* = 24)	2 (8.33%)	10 (41.67%)	9 (37.50%)	3 (12.50%)	0 (0.00%)	0 (0.00%)	
Obese (*n* = 20)	0 (0.00%)	6 (30.00%)	8 (40.00%)	3 (15.00%)	3 (15.00%) *	0 (0.00%)	

* Post hoc test for differences in proportions is significantly higher as compared to other group(s). The *p*-values of the post hoc comparisons for BMI and waist circumference groups were Bonferroni-adjusted. BMI body mass index.

## Data Availability

The data presented in this study are available on request from the corresponding author.
